# Discrimination in Filling Materials

**Published:** 1894-12

**Authors:** J. L. Davenport

**Affiliations:** New York.


THE
AMERICAN JOURNAL
—OF—
DENTAL SCIENCE.

VOL. XXVIII. BALTIMORE, DECEMBER, 1894. No. 8.

ARTICLE I.

### DISCRIMINATION IN FILLING MATERIALS.

DR. J. L. DAVENPORT, NEW YORK.

The question, "What is the best material with which to fill teeth?" has been so often asked and answered, that doubtless many who read the first lines of my article will turn over the leaves in contempt. At a meeting of the Odontological Society, of New York, some years since, this subject was being discussed by many of the able men in the profession, when the President called on Dr. Benjamin Lord for his views. I remember well the sensation when that able, gray-haired veteran arose, and, slowly repeating the question, said: "My opinion is, that the best material with which to fill teeth is *judgment*." The slight shock on those who had been extolling this and that material, was so great that the subject was soon passed. The significance of Dr. Lord's answer was so overwhelmingly logical that the Society members all admitted that the question had been soundly answered.

It is surprising to me, however, that there so few dentists who have benefitted by Dr. Lord's logic.

In view of the many dental colleges, and the vast amount of dental literature flooding the land, it is surprising to see the stubborn ignorance that exists among both the masses and average dentists, as to how best to treat the teeth for their best preservation.

I well remember the bomb thrown into the ranks of the profession by J. Foster Flagg, at a meeting held at the house of Dr. Haws, in New York, once: The teachings of Dr. S. B. Palmer, of Syracuse, N. Y., and Professor Chase, of St. Louis, who, with Dr. Flagg, were the promulgators of what was styled the "New Departure Theory," which was diametrically antagonistic to the accepted practice, which had existed since the days of Drs. Greenwood and Washington. It is true that some of the teachings, by stubborn, old-time professors in the colleges, have been rather antagonistic to the "New Departure Creed," and, consequently, students have not been instructed to exercise that judgment which is so requisite to the modern dentist. To pound gold in soft and frail teeth, for children, with the admonition that it is permanent, is practice of the most reprehensible character. I find amalgam and gold is the standard filling by dentists in the rural towns, with but little regard as to the age of the patient or the quality of the teeth. Oxiphosphate is also largely used by these men, under its various names, conveying to the patient the statement that it is "insoluble." This is nothing less than downright deception. It is true the packages of this material are marked insoluable, but if there has ever been any manufactured that was so, we have never discovered it. It is the most deceptive of all the materials used, as the oral fluids wash it out quickly near the gum, while the remainder of the cavity is comparatively good. I have seen it last for many years on the crowns of molars, when in the same mouth it would melt away from the cervical wall, in proximal and buccal cavitied, in a few months. I remember, when I first began practice, I was taught to believe that all failures were caused by defective manipulation.

This belief we, of course, conveyed to patients; but how sadly they have been disappointed. The masses have but little confidence in dentistry—I mean that dentistry that claims to save teeth. For this state of affairs no one is so much to blame as the dentist himself.

If students were taught to use gutta-percha more and gold less in teeth of young persons, and of a frail character, that confidence so much needed by the dentist would be restored. To do this, however, they must learn to successfully fill with it. Careless manipulation and hurried operations will not suffice. My method is to separate the teeth, either with cotton or tape, for a day or two, before the sitting is given to fill. I then adjust the rubber-dam, after which a Perry separator is gently put in place, turning the screws slowly so as to give neither pain nor fear. (I am speaking now of children who, perhaps, have never before been to the dentist.) Prepare the cavities as carefully as you have been taught when introducing gold. Retaining points, however, if used at all are unnecessary with gutta-percha. I then touch the cavity with copal ether varnish, allowing a few seconds for the evaporation. This is so thin and transparent that it can not be seen in the cavity, but its surface will be a little sticky assisting materially in holding the first pieces of gutta-percha in position. This, of course, should be of good quality. Great care should be taken to not over-heat the gutta-percha, as it becomes brittle. I usually pack with a warm instrument, but just the requisite heat is required to prevent it from sticking. A cold burnisher is held on the filling till it is cool, and then it should be as carefully trimmed as you would finish gold. This work, if the cavities are small and the closing of the space obtained hides them, thus preventing attrition or wear in mastication, will give most marvelous results. In no case, however, is the patient to be told that the work is permanent and will last a lifetime, but must be instructed to have them examined every six months or a year. I do not mean that such work will need renewing in that time, but the fault heretofore has been that dentists have given patients the assurance that their operations were everlasting, and that is why we suffer by the lack of confidence the masses have with us. One great fault with the average dentist is that he has looked on gutta-percha as temporary and little better than wax. He has had no confidence himself with this material as a filling, and consequently has not made a charge for it, equal to its worth. Dr. Flagg stated in his address before the Odontological Society, that his charges or earnings when manipulating the plastics were, or should be, equal to the best gold operators. Claiming, of course, the shorter time consumed in favor of the former materials and the greater good conferred on the greater number thus bringing dentistry to that standard of excellence by which the poor as well as the rich could be benefitted.

The *Items* is everlastingly hammering at the sluggards in the profession, and I hope its good advice may bear fruit. We are such a bigoted, headstrong lot, however, unwilling to learn or investigate, that the editor of the *Items*, I fear, will go under before he can make a respectable lot out of us. No amount of talk or teaching will make good dentists of those who know it all. A man can never get too old to learn. Then there are the dirty and untidy, with no order or system in their offices. I have often remarked that I could judge a dentist by a glance into his laboratory or operating case. If his instruments are rusty and dull, his operations will show like conditions.

The reader must not take this article as advocating the exclusive use of gutta-percha or any particular filling material. They all are best it their proper places. Proximal gold fillings in the teeth of the requisite texture and quality will last many years. It is the want of proper discrimination, and the continued belief of many patients, that going to the dentist once is to finish the job for all time. The fault is with the dentist; we have talked for years about "permanent" fillings. I think the word should be erased from our vocabulary. Gutta-percha, undoubetdly, is not as easily put in as the "insoluble"(?) oxiphosphate or amalgam, but its insolubility is so wonderful we might say it is almost permanent. The President of the Postal Telegraph Company, I do not recall his name, has recently said, in reference to the coating of the commercial Atlantic cable which has just been laid, that the gutta-percha is a protection to the cable lying in the bed of the ocean was "absolutely indestructable." The knowledge that we have a substance of this kind, non-conductive, non-irritant, compatable with tooth bone, thus causing no electrical action, it having already been tested in the mouth sufficient to prove its superior qualities, and as the able gentlemen who originated the "New Departure Creed" have said, "It is the most permanent filling material we possess," to deprive our patients of the benefit of this material is not to do our best for the preservation of their teeth. The cost of this material to the dentist is comparatively insignificant. In England it is cheaper than it is here, as it goes into that country free of duty. This is one reason why all of our long ocean cables have been made in London, as the insulation takes large quantities of it. It is to be hoped that dentists will stop telling their patients of permanent fillings. This is the one great cause why so few of the population patronize the dentist. A man sends his son or daughter to have their teeth put in order; his idea is that the permanent work will at least bear some claim to the name, but in two or three years he finds that the teeth have actually melted away from the beautiful gold jewels that were inserted and for which he paid so highly. He still may have courage, but he condemns that dentist and seeks another who, if he be a mercenary idiot, will condemn the former work, though his own skill as a gold manipulator is perhaps far inferior. The work done by this one also fails. Now, is it a wonder that the people have no confidence in dental operations? The fault lies with the dentist. We do not instruct our patients rightly. Improvement is the practical watchword of the age; let us break away from the theories and beliefs of the past, especially those that have betrayed our confidence and not stood the test.—*Items of Interest.*

